# Treatment of non-epileptic episodes of anxious, fearful behavior in adolescent juvenile neuronal ceroid lipofuscinosis (CLN3 disease)

**DOI:** 10.3389/fneur.2023.1216861

**Published:** 2023-09-12

**Authors:** John R. Ostergaard

**Affiliations:** Department of Child and Adolescence, Centre for Rare Diseases, Aarhus University Hospital, Aarhus, Denmark

**Keywords:** neuronal ceroid lipofuscinoses, JNCL, CLN3, autonomic nervous system, heart rate variability, paroxysmal sympathetic hyperactivity, fear

## Abstract

**Background:**

Recurrent non-epileptic episodes of frightened facial and body expression occur in more than half of post-adolescent patients with juvenile neuronal ceroid lipofuscinosis (JNCL, CLN3 disease). Clinically, the episodes look similar to the attacks of paroxysmal sympathetic hyperactivity (PSH) commonly seen following traumatic brain injury (TBI). The episodes occur when the patients are exposed to separation, hear loud sounds or are otherwise bothered by discomfort and as in PSH following TBI, the attacks are difficult to prevent and/or treat.

**Aim and methods:**

Based on present knowledge of triggering factors, the neural anxiety/fear circuit, its afferent and efferent pathways and documented CLN3 disease-impact on these tracks, the current study discusses a rational approach how to prevent and/or treat the attacks.

**Results:**

Patients with JNCL have a disturbed somatosensory modulation leading to a reduced threshold of pain; a degeneration within the neural anxiety/fear circuit leading to an imbalance of central network inhibition and excitation pathways; and finally, an, with advancing age, increasing autonomic imbalance leading to a significant dominance of the sympathetic neural system.

**Discussion:**

Theoretically, there are three points of attack how to prevent or treat the episodes: (1) increase in threshold of discomfort impact; (2) modulation of imbalance of central network inhibition and excitation, and (3) restoring the balance between the sympathetic and parasympathetic neural systems prompted by a parasympathetic withdrawal. As to (1) and (2), prevention should have the greatest priority. As regards (3), research of transcutaneous vagal stimulation treatment in JNCL is warranted.

## Introduction

Juvenile neuronal ceroid lipofuscinosis (JNCL, CLN3 disease) is a neurodegenerative disease with an incidence of 2–5 per 100,000 (1, 2). Debut occurs at 5–7 years of age, most often as a rapid progressive loss of vision leading to blindness within 2–4 years. The disease propagation continues with loss of cognition, speech, motor abilities, and development of epilepsy and behavioral challenges. Death occurs in the early thirties (3). Typical time course relative to onset of specific clinical symptoms or milestones appears in Table 1. In addition to epilepsy, recurrent episodes of an anxious, fearful behavior, which occur in more than half of post-adolescent JNCL patients and may be related to premature death (4), cause great parental concern (5). As can be seen from Table 1, it is a relatively late symptom of the disease. In addition to a frightened facial appearance while clinging to the bed or chair, the young JNCL patients demonstrate increased atypical muscle activity, excessive sweating, increased body temperature and blood pressure, tachycardia, tachypnea and often show non-sustained multi-directional nystagmus, pupil dilation and a decreased awareness (4–7). The episodes might start without obvious reason, but typically the episodes occur when the patients are being left alone by their caregivers, or are exposed to stimuli that are either non-nociceptive (lifting the patient against gravity, i.e., from bed to a chair/wheelchair) or only minimally nociceptive (bathing, brushing teeth) or when they hear loud sounds (4–7). The episodes initially last for seconds or minutes and occur often in clusters. The rate of episodes increases during the later stages of the disorder and may last up to hours and days (4–7). Phenotypically, the episodes thus resemble the seizures which normally are seen following acute traumatic brain injuries (TBI), known as paroxysmal sympathetic hyperactivity (PSH), and believed to be caused by a disconnection syndrome between sympathetic inhibitory regions (insula, cingulate cortex) and sympathetic activating centers (hypothalamus and brainstem) (8–14).

**Table 1:** Typical time course relative to onset (years in average ranges) of specific clinical symptoms or milestones in patients with juvenile neuronal ceroid lipofuscinosis (CLN3 disease).

Average age range (years) at onset	Clinical symptoms/clinical milestone
5–6	Parental suspicion of vision loss
6–8	Cognitive decline leading to dementia, starting with impairment of attention, short memory and general reasoning abilities
6–8	Behavioral impairment (withdrawn or inattentive behavior; uncooperative and ritualistic disruptive behavior; engaged in repetitive questioning or discussions)
7–9	The diagnosis is made
8–10	Sleep disturbances (settling problems, nocturnal awakenings and nightmares)
9–11	Legal blindness
10–12	Epilepsy (focal or generalized tonic–clonic seizures)
11–14	Hallucinations (often visual, and of a frightening nature: seeing snakes, lizards, lots of ants and flies)
11–17	Speech impairment (articulatory dysfunction and increasing dysfluency)
12–15	Parkinsonian walking (rigidity, bradykinesia, slow steps with flexion in hips and knees, and shuffling gait)
14–20	Cardiac involvement (sick sinus syndrome leading to progressive bradycardia)
15–22	Loss of independent walking (unable of walk without assistance or daily use of wheelchair)
16–20	Bladder and bowel dysfunction
17–24	Recurrent episodes of an anxious, fearful behavior
18–27	Need for enteral feeding tube (percutaneous endoscopic gastrostomy)
22–30	End of life

The attacks develop in the late teens during a phase of the disease where the cognitive developmental age of the affected patients is around, or even below 2 years of age (15); i.e., at a mental developmental age when individuals do not have the cognitive ability to encounter a normal anxiety response. Instead, the anxious behavior has to be perceived as an exaggerated form of a developmental “natural fear”-response, i.e., the fear similar to what happens when healthy toddlers are left alone, feel discomfort or are exposed to stimuli as loud sounds, unknown person or start rising from the ground (16–18). Based on present knowledge of triggering factors, the neural anxiety/fear circuit, its afferent and efferent pathways and the documented CLN3 disease-impact on these tracks, the current study presents and discusses a rational approach and strategy how to treat and/or prevent the attacks.

## Search strategy and selection criteria

PubMed was searched for articles written in the English language and published between Jan 1, 1970, and April 1, 2023 using the search terms “ceroid,” “lipofuscin” “Batten,” “juvenile NCL,” “NCL,” “CLN3,” “neurodegeneration”; “Parkinson’s disease”; “dementia”; “Alzheimer”; “anxiety”; “fear”; “autonomic dysfunction”; “dysautonomia”; “paroxysmal sympathetic hyperactivity”; “sympathetic nervous system”; “parasympathetic nervous system”; “heart rate variability”; “vagal stimulation”; “heart and brain.” The final reference list was generated on the basis of relevance to the topic covered.

## The neural anxiety/fear circuit

Clinical as well as preclinical research suggests that anxiety and fear emerge from alterations in a set of highly interconnected neural circuits, of which the amygdala, ventral hippocampus, and medial prefrontal cortex are key nodes (17–19).

### The input site of the system (the afferent pathway)

**Figure 1:**
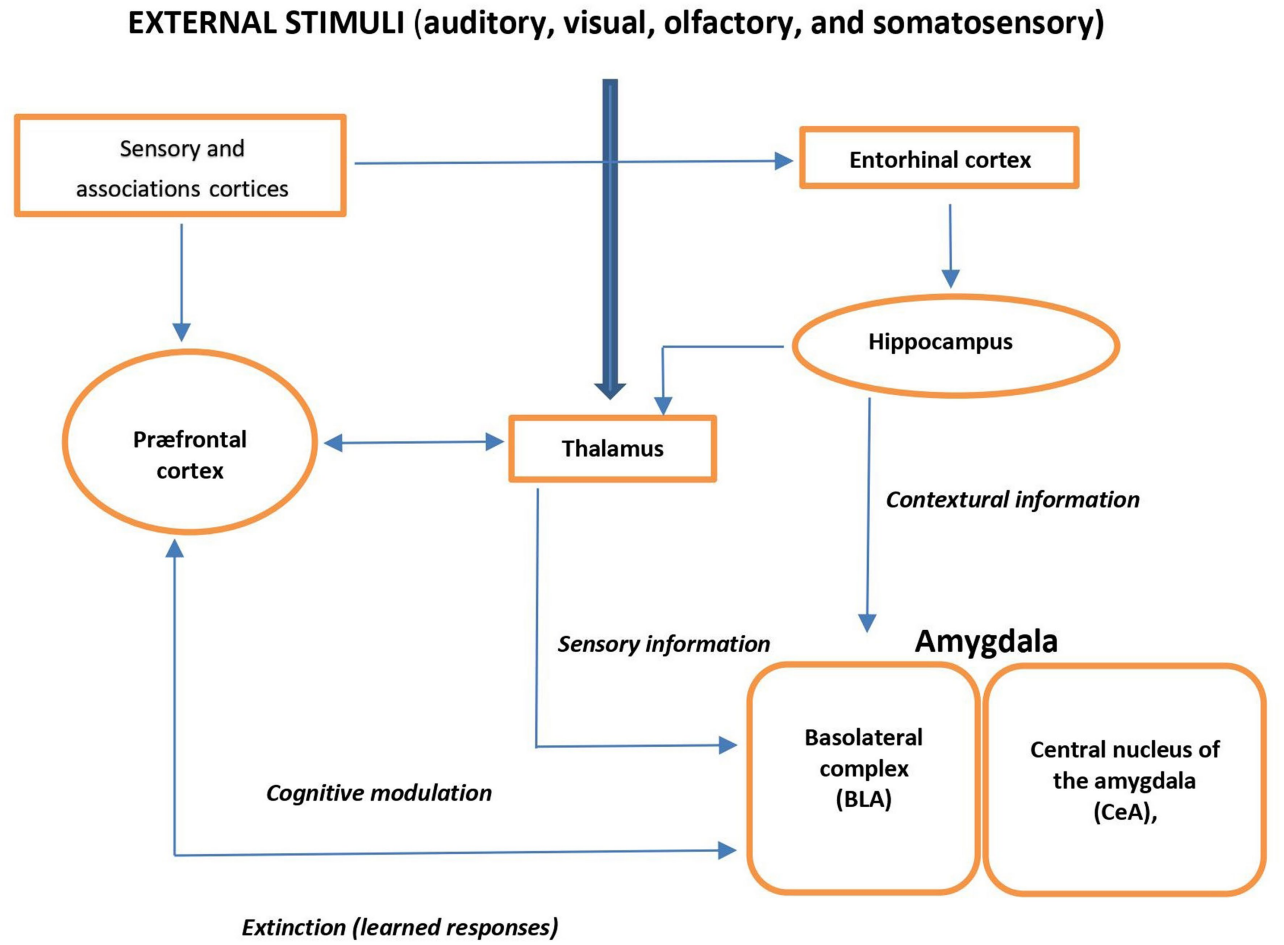
Schema view of major brain circuits involved in fear and anxiety; the input side. External auditory, visual, olfactory, or somatosensory stimuli are relayed by the thalamus to the amygdala and cortex. The basolateral complex (BLA) of the amygdala is the input side of the system, which also receives contextual information from the hippocampal formation (entorhinal cortex, hippocampus, and ventral subiculum). The prefrontal cortex (PFC) processes elaborate “cognitive” information; it modulates the physiological, neuroendocrine, and behavioral responses (*via* the amygdala), and it is also involved in the extinction of fear- and anxiety-related conditional responses.

External auditory, visual, olfactory, or somatosensory stimuli are forwarded through the thalamus to the basolateral complex of the amygdala (BLA) and cortex. BLA receives processed information from the hippocampal formation. *Via* amygdala, the prefrontal cortex (PFC) elaborates cognitive information and modulates the physiological, neuroendocrine, and behavioral responses and is also involved in fear- and anxiety-related conditional responses. Thus, the amygdala plays a central role of the input site of the neural anxiety-fear neural circuit (Figure 1) (17).

### The output site of the system (the efferent pathway)

**Figure 2:**
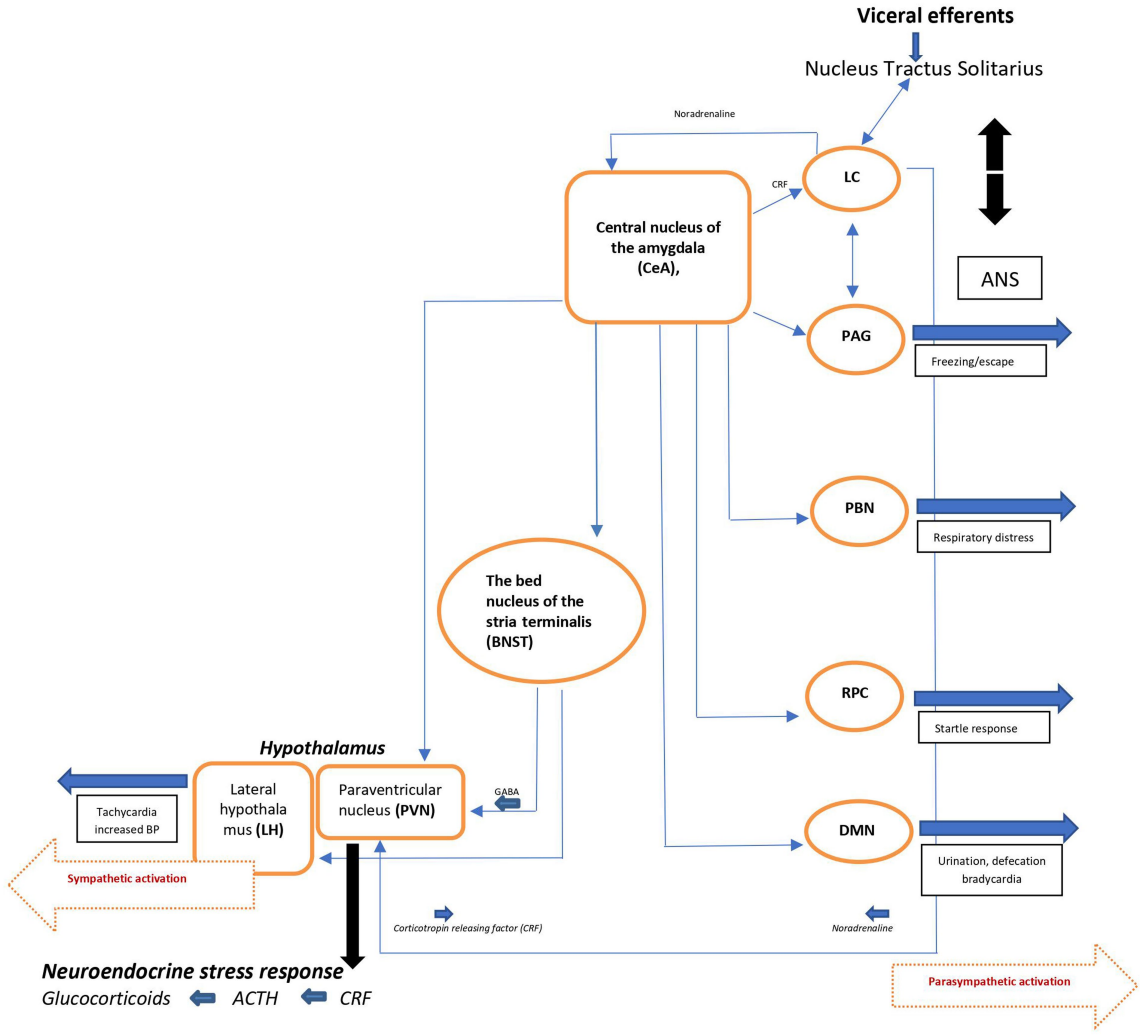
Schematic view of major brain circuits involved in fear and anxiety; the output side. After intra-amygdala processing of the emotional stimuli, the central nucleus of the amygdala (CeA), on the output side, activates the locus ceruleus (LC) and central and peripheral noradrenaline systems [*via* corticotropin-releasing factor (CRF) neurons], and the hypothalamus [paraventricular nucleus (PVN) and lateral hypothalamus (LH)]. The bed nucleus of the stria terminalis (BNST, part of the “extended amygdala”) is also a control center for the neuroendocrine system by integrating information originating from both the hippocampus and the amygdala. In addition, the CeA directly activates various midbrain regions or nuclei responsible for different aspects of the fear/anxiety response: freezing or escape [periaqueductal Gray (PAG)], increased respiratory rate [parabrachial nucleus (PBN)], startle [caudal reticulopontine nucleus of the reticular formation (RPC)], and the dorsal motor nucleus of the vagus (DMN) in the medulla, which (together with the lateral hypothalamus) is responsible for the increase in heart rate and blood pressure associated with emotional events. ACTH, adrenocorticotropic hormone; ANS, autonomic nervous system; BP, blood pressure; NTS, nucleus tractus solitarius.

The efferent pathways of the anxiety-fear circuit are mediated through autonomic, neuroendocrine, and skeletal-motor responses. The autonomic activation appears quickly (seconds) and is produced by the sympathetic and parasympathetic neural systems. Activation of the sympathetic system produces increase in heart rate, blood pressure, and excessive sweating. Through the parasympathetic division, the heart rate and metabolic demands are suppressed. The neuroendocrine axis triggers or facilitates catecholamine and neuropeptide release. The skeletal muscle response is more complex, depending on whether subtle movements involving a few muscle groups of the facial muscles are essential or whether freezing of the body or escape and fight is required (Figure 2) (17, 20).

Since the immediate consequence of autonomic activation is tachycardia, the ascending noradrenergic (NE) system originating from the locus ceruleus (LC) is believed to be the main core of the anxiety-fear response modulation (for details, see ref. 17). The LC-NE system modifies different brain functions, including fear conditioning (21) through release of NE into widely distributed brain areas like the neocortex, hippocampus, thalamus, subthalamic nucleus, and substantia nigra. Some LC neurons extend to the paraventricular nucleus (PVN) and activate the hypothalamo-pituitary-adrenocortical (HPA) axis, facilitating the stress response seen in during anxiety and/or fear. Noradrenergic neurons also project to amygdala, the prefrontal cortex, the bed nucleus of the stria terminalis (BNST), which by integrating information from the hippocampus and the amygdala, also is a control center of the neuro-endocrine system. In addition, the CeA (central nucleus of amygdala) activates various midbrain regions or nuclei responsible for different aspects of the fear/anxiety response like freezing or escape (periaqueductal gray [PAG]). By activating the dorsal motor nucleus of the vagus (DMN) in the medulla, CeA also participates in the regulation of heart rate and blood pressure associated with the fearful emotional events.

## Known impact of CLN3 disease on the input pathways

In addition to the lack of visual stimuli and the resulting difficulties it entails, patients with JNCL have a disturbed somatosensory modulation leading to a reduced threshold for pain; an allodynia (22). Since repeated skin stimulation shows increase in responsiveness and an increased thalamocortical excitability has been demonstrated in the sensorimotor cortex of JNCL patients (23), the pain most probably is of a central rather than a peripheral origin (22).

## Known impact of CLN3 disease on the neural anxiety/fear circuit

So far, there exists no human imaging, histological, or functional studies with a specific focus on the neural anxiety/fear circuit in JNCL. Recently, a top-down CLN3 disease propagation has been proposed (24), with starting point in the internal layers of the retina followed by a secondary spreading through the connectome to the hemispheres, the brainstem, and *via* medulla to the peripheral nerves. Accordingly, conventional magnetic resonance imaging (MRI) studies have shown a progressive cerebral, cerebellar, and hippocampal atrophy (25–27), whereas functional studies using positron emission tomography (PET) and Single photon emission computed tomography (SPECT) in adolescent and adult JNCL patients have demonstrated thalamic, nigrostriatal and striatal dysfunction as well (28, 29). Using a Cln3 knockout (Cln3^Delta-ex1-6^) mice model, an increased anxiety-related behavior has been demonstrated in older mice (30). Grünewald and co-workers suggested that the co-occurrence of severely affected inhibitory and excitatory synaptic transmission in the amygdala, hippocampus and loss of GABAergic interneurons thereby fosters an imbalance of network inhibition and excitation (30). The present studies thus point to changes due to degenerative processes in CLN3 disease that also are believed to be important in the patho-etiology of PSH following TBI (5, 13, 14).

## Known impact of CLN3 disease on the output pathways

The efferent pathways of the anxiety-fear circuit include autonomic, neuroendocrine, and skeletal-motor responses. As described, activation of the autonomic nervous system occurs fast, and since the immediate consequence is an increase in heart rate, an important pivot must be the balance between the activating sympathetic system and the inhibiting parasympathetic system. Variations of heart-rate variability (HRV) can be used as a proxy for the activity of the autonomic nervous system (5, 31). In JNCL, a significant age-dependent decrease of the parasympathetic activity has been documented without any accompanying age-related change of the neural and neuro-hormonal sympathetic activities (5, 31). Beyond 18–20 years of age the activity of parasympathetic activity is very low (5), and when an adolescent or adult JNCL patient, having a developmental cognitive level equal to a 1–2-year-old child, experiences the sensation of fear, the markedly autonomic imbalance with a dominance of sympathetic activity leaves the ascending noradrenergic system originated from the locus ceruleus (LC) without its normal parasympathetic counterbalance, the fear response will be exaggerated in relation to the stimulus at hand.

The lack of increase in the neural and neuro-hormonal induced sympathetic activity in JNCL with increasing age (5, 31) indicates that a major stimulating role of neuro-endocrine pathways of the anxiety/fear circuit is weak or absent. However, so far, there exist no investigations of the adrenergic hormone or transmitter levels during the attacks in JNCL patients, and therefore a neuro-endocrine impact cannot be totally excluded. In PSH following TBI, median values of the Adrenocorticotropic Hormone (ACTH) and cortisol concentrations are moderately elevated whereas the neurotransmitter concentrations (noradrenaline, epinephrine, dopamine) are significantly increased to levels that nearly equalize the increase regarded as diagnostic in patients with suspected pheochromocytoma (32). Although some of the clinical symptoms of pheochromocytoma (palpitations, diaphoresis and paroxysmal hypertension) are identical to the episodic fearful symptoms in JNCL, others are quite different, like for instance paleness in form of a blotchy or mottled skin color which is a common symptom of pheochromocytoma and caused by adrenergic constriction of the skin blood vessels. In JNCL, on the contrary, the skin is warm, red, and flushing. Anxiety and muscle activity, especially tremor, can be seen in patients with pheochromocytoma, but they do not have an anxious behavior or atypical muscle activity of the same high intensity as in JNCL.

Heart rate variability studies in PSH related to TBI have shown both a higher sympathetic excitatory activity and a lower parasympathetic activity in patients with TBI when compared to controls in general (no TBI), and that during several months after their brain injury. Of note, no differences in heart rate variability were found when TBI patients with and without symptoms of PSH was compared (33, 34). In PSH following TBI, the attacks may even occur when the patients are still sedated and unable to feel fear. In addition, in the majority of TBI patients, the episodes are transient and is phased out within 1–2 weeks (35). In others it persists several months, is related to severity and propagation of the brain damage and a bad prognosis (12–14). In JNCL, the attacks disappear when the patients sleep or are sedated, worsen in frequency, length and often also in intensity over the years. Additionally, in JNCL the sympathetic-parasympathetic imbalance seems more related to a severe decrease in the parasympathetic neural activity than an increase in the neuro-humeral sympathetic activity demonstrated in TBI (33, 34). Therefore, despite many phenotypic similarities, including the allodynia, atypical motor activity and seizure-like appearance there may nevertheless be individual patho-etiological autonomic differences between the well described PSH following TBI and the PSH-like, non-epileptic episodes seen in adolescent and adult JNCL patients.

The key node of the regulatory control of the skeletal muscle is the central nucleus of the amygdala (CeA) (17, 20), which, by mingling of the emotional processing and the balance between the sympathetic and parasympathetic activity, stimulates various midbrain regions or nuclei responsible for different aspects of the fear/anxiety response, including skeletal muscle responses like freezing or escape, startling and respiratory movements. In JNCL, the motor activity is primarily an increase in atypical motor activity, including shivering and a dystonic positioning similar to the abnormal motor activity seen in PSH following TBI (13, 14).

In summary, patients with JNCL have a disturbed somatosensory modulation leading to a reduced threshold of pain; degeneration within the central neural anxiety/fear circuit leading to an imbalance of the central network inhibition and excitation pathways; and finally, an, with advancing age, increasing imbalance between the sympathetic and parasympathetic neural activity resulting in a significant dominance of the sympathetic branch beyond early adolescence.

Theoretically, there are thus 3 points of attack when trying to treat or prevent the episodes: (1) increase in threshold of discomfort impact; (2) modulation of the imbalance of central network inhibition and excitation, and (3) restoring the balance between the sympathetic and parasympathetic neural systems. In relation to the latter, it is important to note that low parasympathetic activity by itself is accompanied by increased anxiety readiness in children (35) and that low parasympathetic activity decreases nociceptive sensitivity and is accompanied by an increase in visceral pain and a generalized somatosensory allodynia (36). A restoring of the sympathetic-parasympathetic balance may thus have a dual effect.

## Modes of treatment

### Increase in threshold of somatosensory pain and discomfort

As described do patients with JNCL have a disturbed somatosensory modulation leading to a generalized reduced threshold of pain (22). Thus, prevention or minimizing the discomfort/pain should have the greatest priority. Baguley and coworkers reported that Gabapentin, which is often used to reduce neuropathic pain, had a positive preventing effect (37). In order to reduce all different kinds of possible discomfort, it is important also to address fecal impaction, urine retention, dehydration, infections, and unfamiliar situations. Daily routines should be as steady and predictable as possible, and it is important to ensure an appropriate positioning and a close but calming physical presence of caregivers. Emotions are “contagious, i.e., can spread from one person to another, and do that through a physiological synchrony between a “demonstrator’s” and the “observer’s” autonomic nervous system (38). Therefore, an important preventive approach is that the caretakers keep their own concerns of the patient’s clinical condition for themselves when the patients are nearby. Besides pain and general discomfort, the episodes occur particularly in situations that are difficult to avoid in connection with handling of young people and adults with JNCL, i.e., necessary general nursing care like bathing, brushing teeth, toilet visits and use of lifts when moving around. In humans, it has long been acknowledged that fear conditioning is related to an increase in parasympathetic activity (35). Thus, a long-term, but early initiated plan for handling the general care measures situations will, through habituation and conditioning and the attendant increase in parasympathetic activity, probably reduce the fear induced by these situations. Theoretically, the increase in parasympathetic activity might also increase the threshold of the generalized somatosensory allodynia (36).

### Modulation of the imbalance of central network inhibition and excitation

The imbalance of inhibition and excitation within the core of the neural fear circuit, i.e., the amygdala, ventral hippocampus and medial prefrontal cortex, is especially related to glutaminergic and GABAergic transmissions. Accordingly, in the Cln3^Dex1-6^ mice model of Grünewald and coworkers (30), an age-dependent significant increase in anxiety-like behavior were associated with a defective synaptic transmission and loss of GABAergic interneurons within the anxiety-fear circuit. No similar or specific histological studies exist in humans. However, consecutive human MRI studies in JNCL patients have shown an age-dependent selectively marked decrease in precisely the hippocampal size (26).

To date, none of the recommended pharmacological treatment schedules of PSH are supported by solid evidence. Clinical experience from treatment in TBI, however, supports the value of benzodiazepines (midazolam, clonazepam) and potent analgesia (10, 13), endorsing the importance of a defective glutamatergic and GABAergic transmission behind the fearful behavior. Worth to note is that dopamine antagonists are either without effect, cause serious side effects, or may even worsen the symptoms (37, 39).

In Table 2, a schedule of theoretically possible treatment issues related to the three different causation levels: (1) increase in threshold of discomfort impact; (2) modulation of the imbalance of central network inhibition and excitation; (3) restoring the balance between the sympathetic and parasympathetic neural systems, is presented, as is a possible method of symptomatic treatment of the present tachycardia, hypertension, and diaphoresis. These data are provided from published reports of drugs used to treat patients with PSH following traumatic brain injuries [for review, see ref. (13)], and not as a 1:1 recommendation for treatment in CLN3 patients as, so far, no treatment trials in CLN3 disease have been published and/or been performed.

**Table 2:** A schedule of theoretically possible modalities for prevention and treatment of the paroxysmal sympathetic hyperactivity (PSH)-like episodes related to the different points of attack.

	Evidence of efficacy^#^^,^^*^
*Increase in threshold of somatosensory pain and discomfort (PREVENTION)*	
Gabapentin: 100 mg every 8 h, orally; titrate to a maximum of 3,600 mg/day	Consistent
Fentanyl: patch 12–100 μg/h	Consistent
*Modulation of the imbalance of central network inhibition and excitation (PREVENTION)*	
Clonazepam: 0.5–8.0 mg/24 h, orally	Consistent
Baclofen: 5 mg every 8 h, orally; titrate to a maximum of 80 mg/day	Limited
Intrathecal—specialist use only	Consistent
Propofol: intravenous infusion; maximum <4 mg/kg per hour	Consistent
*Modulation of the imbalance of central network inhibition and excitation (TREATMENT)*	
Diazepam: 1–10 mg intravenous bolus	Intermediate
Lorazepam: 1–4 mg intravenous bolus	Intermediate
Midazolam: 1–2 mg intravenous bolus	Intermediate
Propofol: 10–20 mg intravenous bolus	Intermediate
Morphine: 1–10 mg intravenous bolus	Intermediate
*Restoring the balance between the sympathetic and parasympathetic neural systems (PREVENTION and TREATMENT)*	
Transcutaneous auricular vagal nerve stimulation (tVNS)	Current lack of knowledge about optimal pulse width, frequency and intensity
*Symptomatic treatment of tachycardia, hypertension, and diaphoresis (PREVENTION)*	
Propranolol: 20–60 mg every 4–6 h, orally	Consistent

The data are provided on the basis of modalities used for PSH due to acute traumatic brain injuries and not from studies in CLN3 disease.

# These data are provided from published reports of drugs used to treat patients with paroxysmal sympathetic hyperactivity (PSH) following traumatic brain injuries (TBI), and not as recommendations for treatment in CLN3 patients as, so far, no treatment trials in CLN3 disease have been published.

* Evidence of efficacy is described as consistent when many or most of the accessible publications showed benefits; intermediate when there was an equivocal impression of benefit in the literature; limited when data were scarce and inconclusive but showed some benefit, or ineffective when the literature showed no benefit. These data are provided from published reports of drugs used to treat patients with paroxysmal sympathetic hyperactivity (PSH) following traumatic brain injuries (TBI).

### Restoring the balance between the sympathetic and parasympathetic neural systems

A clear distinction between a sympathetic hyperactivity related to the actual fear exposure and an eventually autonomic dysfunctions due to the neurodegenerative process itself is difficult to clarify, and the demonstrated autonomic dysfunction may be an epiphenomenon not causative related to the fear response. However, in the literature there are plenty examples of a relationship between an autonomic dysfunction and pathological clinical phenomena in other neurodegenerative diseases. In Huntington’s disease, an increased sympathetic activity, as detected by HRV measures, is associated with repeated episodes of falling unrelated to orthostatic phenomena (40), and autonomic failure in Parkinson’s disease is accompanied by a higher incidence and level of anxiety (41). Recently, a characteristic autonomic response consisting of a parasympathetic withdrawal followed by a sympathetic activation has been demonstrated during outbursts of combative behavior in patients with vascular and Alzheimer dementia (42). Additionally, recent studies have shown that in children a well-functioning vagal alertness is mandatory for coping strategies of fear learning and fear extinction (43).

Theoretically, stimulation of the vagal nerve might be able to restore the sympatho-vagal imbalance (44). Invasive cervical vagal nerve stimulation (iVNS) has been used in >50 years and is approved for the treatment of severe epilepsy, depression, obesity, and for stroke-rehabilitation (45). Possible adverse events, including the invasiveness, restricts its use for research purposes. Transcutaneous stimulation (tVNS) of the cymba conchae of left auricular branch is considered an effective non-invasive alternative (45–47) and has been used in patients with epilepsy, depression or anxiety disorders, and has been tested in Alzheimer’s and Parkinson’s diseases (for review, see ref. 46). Side-effects are minor and mainly include skin reddening and irritation (48). In healthy, auricular tVNS decreases the Low Frequency: High Frequency ratio of HRV, and as simultaneous micrographic recordings reveal a significant decrease in frequency and incidence of muscle sympathetic nerve activity, tVNS seems to be able to increase the parasympathetic activity as well as to reduce the firing of sympathetic fibers (49). However, the efficiency of auricular tVNS depends to a large extent on optimization of critical stimulation parameters like pulse width, frequency and intensity, and the current lack of knowledge about the optimal - ideally individualized - stimulation parameters is a general limitation (50, 51). No case descriptions or trials using invasive or transcutaneous vagal nerve stimulation in the treatment of epilepsy in JNCL patients or in any of the other forms of the neuronal ceroid lipofuscinoses (NCLs) have been published, nor in patients with PSH following TBI.

In summary, treatment and prevention of the episodic attacks should:

1. Focus on minimization of provoking situations and increasing the threshold of somatosensory pain and discomfort; and
2. If and when the episodes occur, modulation of the imbalance of network inhibition and excitation should be countered by use of GABA-nergic drugs and potent analgetica, eventually morphine.
3. Last but not least, since a decrease in parasympathetic activity seems to be the main cause of the sympathetic and parasympathetic imbalance of the output pathways of the anxiety/fear circuit, and also has a great impact of fear conditioning and threshold of a generalized somatosensory allodynia, research of vagal stimulation treatment in JNCL should have a high priority.

## Author contributions

The author confirms being the sole contributor of this work and has approved it for publication.

## Funding

This work was funded by Legacy of Emanuel Poulsen.

## Conflict of interest

The author declares that the research was conducted in the absence of any commercial or financial relationships that could be construed as a potential conflict of interest.

## Publisher’s note

All claims expressed in this article are solely those of the authors and do not necessarily represent those of their affiliated organizations, or those of the publisher, the editors and the reviewers. Any product that may be evaluated in this article, or claim that may be made by its manufacturer, is not guaranteed or endorsed by the publisher.
